# Employment benefits and job retention: evidence among patients with colorectal cancer

**DOI:** 10.1002/cam4.1371

**Published:** 2018-02-23

**Authors:** Christine M. Veenstra, Paul Abrahamse, Todd H. Wagner, Sarah T. Hawley, Mousumi Banerjee, Arden M. Morris

**Affiliations:** ^1^ Institute for Healthcare Policy and Innovation University of Michigan Ann Arbor Michigan; ^2^ Center for Healthcare Outcomes and Policy University of Michigan Ann Arbor Michigan; ^3^ Department of Veterans Affairs Palo Alto Health Care System Health Economics Resource Center Menlo Park California; ^4^ Stanford‐Surgery Policy Improvement Research and Education Center Stanford University School of Medicine Stanford California

**Keywords:** Colorectal neoplasms, employment, insurance, health, sick leave, surveys and questionnaires

## Abstract

A “health shock,” that is, a large, unanticipated adverse health event, can have long‐term financial implications for patients and their families. Colorectal cancer is the third most commonly diagnosed cancer among men and women and is an example of a specific health shock. We examined whether specific benefits (employer‐based health insurance, paid sick leave, extended sick leave, unpaid time off, disability benefits) are associated with job retention after diagnosis and treatment of colorectal cancer. In 2011–14, we surveyed patients with Stage III colorectal cancer from two representative SEER registries. The final sample was 1301 patients (68% survey response rate). For this study, we excluded 735 respondents who were not employed and 20 with unknown employment status. The final analytic sample included 546 respondents. Job retention in the year following diagnosis was assessed, and multivariable logistic regression was used to evaluate associations between job retention and access to specific employment benefits. Employer‐based health insurance (OR = 2.97; 95% CI = 1.56–6.01; *P* = 0.003) and paid sick leave (OR = 2.93; 95% CI = 1.23–6.98; *P* = 0.015) were significantly associated with job retention, after adjusting for sociodemographic, clinical, geographic, and job characteristics.

Although implementation of the Patient Protection and Affordable Care Act (ACA) expanded access to preventive medical care among working Americans who were uninsured or underinsured 1, serious illness still may cause considerable financial burden 2, 3, 4. For a substantial number of patients, bankruptcy due to serious illness or its treatment is a real risk 4, 5. The risk of bankruptcy and attendant financial burden is exacerbated by illness‐ or treatment‐related job loss 6. Employment benefits such as paid sick leave and disability benefits may help working patients retain their jobs during and after serious illness. Among workers who are eligible, the Family Medical Leave Act (FMLA) provides protection for short‐term disability in the form of unpaid leave and has been shown to influence job retention among new mothers 7.

The provision of paid medical leave, however, is not mandated under FMLA or the ACA, nor is it a part of standard health insurance coverage 2. In fact, 40% of US workers do not have access to FMLA 8 and up to 60% do not have access to paid sick leave 9. A substantial proportion of workers who experience a “health shock,” that is, a large, unanticipated adverse health event, may therefore lack the employment benefits needed to undergo treatment, recover, and return to work. Additionally, a robust literature has shown the effect of health shocks on labor outcomes 10, 11, 12, 13. These studies frequently use broad proxies for the health shock, such as changes in self‐reported health status or the presence of an inpatient stay. We addressed this issue in more granular detail by focusing on colorectal cancer, a specific health shock.

Colorectal cancer is a prevalent and serious illness frequently diagnosed among working adults without prior warning 14, 15. Treatment for advanced but curable (Stage III) colorectal cancer typically requires both major surgery and chemotherapy, initiated within 4 months of diagnosis and lasting up to 9 months. Cancer treatment can disrupt a working person's day‐to‐day life physically and economically for some or all of this time. Working‐age cancer survivors, who may be supporting young families or repaying educational debt and face a greater loss of potential earnings than older survivors, are more likely to report financial hardship 16. In previous work, we reported that as a result of diagnosis or treatment, over 50% of colorectal cancer patients reduce spending on general expenses, one‐third use savings, one‐third cut down on spending for food or clothing 3, 17, and nearly 50% who are employed at the time of diagnosis are unable to retain their jobs 6. In this study, we used data from a population‐based survey of patients with Stage III colorectal cancer to examine the association of employee benefits (employer‐based health insurance, paid sick leave, extended sick leave, unpaid time off, disability benefits) with job retention.

## Methods

### Study population

We identified all patients ≥18 years of age with surgically resected, pathologic Stage III colon or rectal cancer reported to the Surveillance, Epidemiology and End Results (SEER) cancer registries of metropolitan Detroit and the state of Georgia between 1 August 2011 and 31 December 2014. These sites were selected because of their demographically and racially diverse patient populations. Patients were eligible for study recruitment at 4 months following diagnosis, when all treatment should have been initiated, and returned surveys were accepted up to 12 months after diagnosis. Exclusion criteria included metastatic cancer (Stage IV) at diagnosis, change in diagnosis based on final histology, death prior to survey deployment, or residence outside SEER catchment area. We also excluded respondents who reported no employment at the time of diagnosis.

### Data collection

We notified physicians of our intention to contact study subjects. After an opt‐out period by physicians, patients were invited to participate using a modified multimodal Dillman approach 18. Upon receipt of surveys, we performed extensive data checks for logic, errors, and omissions. We recontacted patients as necessary to obtain missing information and supplemented data with SEER registry‐provided clinical data and census tract‐level socioeconomic status (SES) data through a 2010 Census linkage.

The study protocol was approved by the institutional review boards of the University of Michigan, Wayne State University, Emory University, the State of Michigan, and the State of Georgia Department of Public Health.

### Measures

The primary outcome was job retention at the time of survey. We asked respondents who were employed at the time of diagnosis if they were working at the time of survey completion (mean 8 months later). We validated responses with the additional survey question: “Are you currently working for pay?” (yes/no response). We also asked patients how much work they had missed as a result of cancer or its treatment.

We also explored “job lock”—whether individuals remained in their jobs due to fear that they would otherwise lose health insurance coverage for themselves or their families 19. We assessed this by asking patients whether, as a result of their colorectal cancer diagnosis or treatment, they kept their job mainly to keep their health insurance (yes/no response).

The primary exposure, availability of employment benefits, was assessed by asking respondents whether any of the following were available to them through their employer during cancer treatment: employer‐based health insurance, paid sick leave, extended sick leave, disability, unpaid leave, “other.” Respondents could select all that applied; 83 respondents left one or more of the benefit options blank. They were included in bivariate analyses but excluded from multivariable regression models. Additional covariates included self‐reported patient‐level sociodemographic characteristics: age at diagnosis, gender, race, marital status, level of educational attainment, annual household income, comorbid conditions before the cancer diagnosis, overall health status, availability and type of health insurance (none, Medicaid, Medicare, Private: employer‐based, self‐purchased, and through spouse); area‐level characteristics: geographic region, area‐level socioeconomic status (SES) index (principle component analysis of area‐level high school degree, college degree, poverty level combined into a composite standardized measure of the economic environment); and job‐related characteristics: type of occupation (white collar/blue collar derived from patient‐reported categories of manual, clerical, management/professional, military, self‐employed). The majority (>90%) of employed respondents received adjuvant chemotherapy. Thus, we did not include chemotherapy receipt as a covariate.

There were few missing values (<2%) for all variables except annual household income, for which 19% of patients did not respond or reported they did not know. Multiple imputation techniques were used to account for the missing annual household income data.

### Statistical analyses

Our analytic sample consisted of all survey respondents who were working for pay at the time of cancer diagnosis. We generated descriptive statistics of time away from work due to diagnosis and treatment. We evaluated the association of job retention with patient‐level sociodemographic, area‐level, and job‐related covariates. We then examined the association of job retention with the availability of specified employment benefits. Chi‐squared tests were used to assess bivariate associations. We conducted a sensitivity analysis by constructing a cumulative variable counting the total number of job support benefits available to each respondent (range 0–6). This was tested in each model and yielded similar results to the models that used individual employment benefits. The exclusion of working patients over age 65 did not significantly change any results. Thus, they are included in the study.

To examine the relative impact of sociodemographic, area‐level, and job‐related factors (including employment benefits), on job retention, we used multivariable logistic regression to predict job retention, accounting only for patient‐level sociodemographic and area‐level covariates in the first model. We then added job‐related covariates including employment supports in a second model. Finally, as a sensitivity analysis, we fitted multivariable logistic regression to model job retention as a function of patient‐level sociodemographic and area‐level covariates as well as employment benefits, stratified by type of occupation (blue collar vs. white collar). The results of the stratified analyses were similar to those of the unstratified analysis and are therefore not described in more detail.

To address potential confounding by worker selection into “good jobs” (those more likely to offer employment benefits) and “bad jobs” (those less likely to offer employment benefits), we employed propensity score‐based analysis. We selected the availability of paid sick leave as the indicator variable in our propensity score analysis because it is policy‐relevant and the most tangible of the employment benefits investigated in our studies. Availability of paid sick leave was analyzed as a patient‐reported dichotomous variable (paid sick leave yes/no). In the first step of the analyses, propensity scores were estimated based on a logit model of the paid sick leave indicator variable, given the observed covariates age, race, education, gender, marital status, income, number of comorbid conditions, job category, SEER site, and composite census‐level SES. The propensity score for an individual, defined as the conditional probability of having paid sick leave given the individual's covariates, has been shown to serve as a balancing score and can be used to reduce bias due to any covariate imbalance 20. Once estimated, the propensity scores were grouped into quintiles. Logistic regression was used in the second‐step analysis to model job retention as a function of the availability of paid sick leave and sociodemographic, area‐level, and job‐related factors, including other employment benefits, and propensity score quintiles from the first‐step model. Additional information demonstrating the adequacy of the propensity score analysis is presented in the Statistical Appendix.

We evaluated bivariate associations between the exploratory outcome of job lock and covariates using chi‐squared tests. Wald F‐tests were used to assess associations and multivariable logistic regression for adjusted analyses. All statistical tests were two‐sided. *P* value <0.05 was considered statistically significant. Multicollinearity between covariates was tested using the variance inflation factor, and all pairwise interactions were tested for significance. All analyses were conducted using SAS 9.4 software (Cary, NC).

## Results

### Study sample and response rate

We identified 2168 patients with Stage III colorectal cancer reported to the SEER registries of Georgia and Detroit using Rapid Case Ascertainment. Among these, 259 (12%) were later determined ineligible (metastatic disease, noncolorectal primary, prior cancer diagnosis, residing outside the registry catchment area). Among 1909 eligible patients included in the final sample, 608 could not be located or did not return the survey, leaving 1301 patients (68% survey response rate). For this study, patients who were not working at the time of diagnosis (*n* = 735) and those missing employment information (*n* = 20) were excluded, leaving a final analytic cohort of 546 respondents.

### Overall respondent characteristics and job impact

Among 546 employed respondents, 57% were male, 69% were white, 67% were married, 28% reported high school education or less, 38% reported annual household income less than $50,000, and 33% reported two or more comorbid conditions (Table 1). Eighteen percent of employed respondents were age 65 or older, reflective of broader trends in the US labor force: In 2010, 17.4% of US workers were over age 65 21. As a result of colorectal cancer diagnosis and treatment, 17% of respondents missed <1 month of work, 29% missed 1–3 months, 20% missed 3–6 months, and 17% missed 6–12 months of work.

**Table 1:** Patient sociodemographic and area‐level characteristics and job retention among working respondents

Patient characteristic	*N* = 546	% Retained job	*P*‐value
Sociodemographic			
Age			
<50	164	54	0.759
50–64	285	57	
≥65	96	54	
Gender			
Male	309	62	0.001
Female	233	48	
Race			
White	371	62	<0.001
Black	138	43	
Other	32	38	
Marital status			
Not married/partnered	181	50	0.005
Married/partnered	363	60	
Education			
<High school	48	23	<0.001
High school	104	41	
Some college	194	52	
College graduate	192	74	
Household income^a^			
<$20,000	48	13	<0.001
$20,000–$49,000	136	44	
$50,000–$89,000	161	60	
≥$90,000	139	78	
Comorbid conditions			
0	182	64	0.001
1	182	58	
2 or more	180	44	
Overall health			
Excellent	164	75	<0.001
Good	285	64	
Poor	96	20	
Area‐level			
SEER site			
Detroit	162	53	0.458
Georgia	382	57	
Area‐level SES, tertile			
High	165	71	<0.001
Medium	206	53	
Low	171	44	

a Information about household income was missing for 19% of respondents and was imputed in subsequent multivariate models.

Fifty‐five percent of working respondents retained their jobs. In unadjusted analyses, those who retained their jobs were significantly more likely to be male, white, married, and to report a higher level of educational attainment, higher annual household income, 0 or 1 comorbid conditions, and excellent health (all *P* < 0.01). Those residing in the upper tertile of area‐level SES were also significantly more likely to retain their jobs (*P* < 0.01).

### Employment benefits and job retention

Among working respondents, 70% reported availability of employer‐based health insurance (Table 2). Fifty‐one percent reported availability of paid sick leave, 52% extended sick leave, 46% unpaid time off, and 57% disability benefits. Thirteen percent of respondents reported no health‐related employment benefits. These respondents were more likely to be older, black, self‐employed, and report lower annual household income (all *P* < 0.01; data not shown). Respondents aged 65 and older were significantly less likely to have each of the employment benefits compared with younger respondents.

**Table 2:** Job‐related characteristics and job retention

Patient characteristic	*N* = 546	Retained job (%)	*P*‐value
Job category			
Blue collar	188	35	<0.001
White collar	322	66	
Unclassified/unknown	12	58	
Employer‐based health insurance			
No	163	34	<0.001
Yes	381	65	
Paid sick leave			
No	241	45	<0.001
Yes	253	67	
Extended sick leave			
No	233	51	0.038
Yes	255	60	
Unpaid time off			
No	271	49	0.004
Yes	231	62	
Disability benefits			
No	206	30	<0.001
Yes	278	76	

In unadjusted analyses, those who retained their jobs were significantly more likely to have each of the individual employment benefits (employer‐based health insurance, paid sick leave, extended sick leave, unpaid time off, disability benefits) available. Additionally, in a count of employment benefits, we noted a linear association between the number of available benefits and odds of job retention (*P* < 0.01; data not shown).

### Relative impact of patient‐level sociodemographic, area‐level, and job‐related characteristics on job retention

In the fully adjusted model including individual‐ and area‐level variables (age, education, income, race, gender, marital status, comorbid conditions, overall health, SEER site, and area‐level socioeconomic status) as well as job characteristics, availability of employer‐based health insurance (OR = 2.97; 95% CI = 1.56–6.01; *P* = 0.003) and availability of paid sick leave (OR = 2.93; 95% CI = 1.23–6.98; *P* = 0.015) were significantly associated with job retention. Patients with poor health were less likely than those with good health to retain their jobs (OR = 0.07; 95% CI = 0.03–0.16; *P* < 0.01). While income was significantly associated with job retention in the first model controlling for only sociodemographic and area‐level characteristics, it was no longer significant in the second model that additionally accounted for job‐related characteristics (Table 3). In addition, blue‐collar workers were significantly more likely than white‐collar workers to report “job lock,” that is, that they kept their jobs in order to maintain health insurance (25% vs. 16%; *P* = 0.02). There were no significant differences in job lock by age of respondent.

**Table 3:** Multivariable logistic regression of job retention

	Model 1: Hazard ratio (HR) of job retention by sociodemographic and area‐level characteristics		Model 2: HR of job retention (Model 1 + job‐related characteristics)	
	HR (95% CI)	*P*‐value	HR (95% CI)	*P*‐value
Age				
<50	1.0 (ref)	0.611	1.0 (ref)	0.265
50–64	1.34 (0.74–2.42)		1.18 (0.64–2.16)	
>65	1.34 (0.58–3.10)		2.12 (1.85–5.28)	
Education				
<High school	1.0 (ref)	0.122	1.0 (ref)	0.386
High school	1.80 (0.55–5.87)		1.61 (0.48–5.34)	
Some college	1.70 (0.52–5.55)		1.10 (0.33–3.67)	
College graduate	3.20 (0.93–10.98)		1.86 (0.52–6.60)	
Income				
<$20K	1.0 (ref)	0.002	1.0 (ref)	0.404
$20–$49K	2.85 (1.22–6.68)		1.20 (0.48–3.00)	
$50–$89K	3.81 (1.49–9.75)		1.37 (0.50–3.73)	
≥$90K	7.65 (2.68–21.87)		2.31 (0.72–7.38)	
Overall health				
Excellent	1.0 (ref)	<0.001	1.0 (ref)	<0.001
Good	0.68 (0.35–1.30)		0.61 (0.28–1.31)	
Poor	0.07 (0.03–0.16)		0.06 (0.03‐0.14)	
Job category				
White collar			1.0 (ref)	0.247
Blue collar			0.56 (0.28–1.11)	
Unclassified			0.68 (0.13–3.73)	
Employer‐based health insurance				
No			1.0 (ref)	0.003
Yes			2.97 (1.56–6.01)	
Paid sick leave				
No			1.0 (ref)	0.015
Yes			2.93 (1.23–6.98)	
Extended sick leave				
No			1.0 (ref)	0.335
Yes			1.41 (0.61–2.12)	
Unpaid time off				
No			1.0 (ref)	0.411
Yes			0.79 (0.44–1.40)	
Disability benefits				
No			1.0 (ref)	0.109
Yes			0.55 (0.27–1.14)	

95% CI: 95% confidence interval. SEER: Surveillance Epidemiology and End Results cancer registry; area‐level SES: geographic socioeconomic status based upon census income and education data aggregated at the zip‐code level.

In the preliminary adjusted model that included individual‐ and area‐level variables, only annual household income and overall health were significantly associated with job retention. In the fully adjusted model including job characteristics, income was no longer statistically significant. Both models were also adjusted for race, gender, marital status, comorbid conditions, SEER site, and area‐level socioeconomic status (SES). These covariates were not statistically significant and are therefore omitted from the table.

In the propensity score analysis, paid sick leave remained independently significantly predictive of job retention after adjusting for all other covariates as well as propensity quintile (*P* = 0.02). We did not find significant differences in job retention by gender nor did we find significant two‐way interactions with gender.

## Discussion

Stage III colorectal cancer is a major unexpected illness that can have long‐term financial implications for patients and their families. Among employees diagnosed with Stage III colon cancer, employment benefits designed to ameliorate health shock related to job loss worked as expected. Even after controlling for patient‐level sociodemographic, area‐level, and job‐related characteristics, the availability of employer‐based health insurance and paid sick leave were significantly associated with job retention. Our specific investigation of paid sick leave was robust to multiple sensitivity analyses including adjusting for propensity score quintiles and stratifying the multivariable logistic regression model by type of occupation, supporting a causal relationship irrespective of individual, area‐level, and job characteristics.

While job retention did not vary significantly between blue‐collar and white‐collar workers, blue‐collar workers were significantly more likely to endorse “job lock” phenomenon; 25% reported that they kept their jobs in order to keep their health insurance. These findings highlight the vulnerability of some patients based on occupation, the complexity of employment impacts of health shocks, and the nuanced role health insurance plays in patients’ employment decisions and financial well‐being. While insurance coverage concerns may have been partially alleviated by the Affordable Care Act, as insurance options in the United States continue to evolve, it will become even more important to understand the role of insurance and employment in the face of a health shock and for clinicians and policy makers to recognize not only who is at risk for job loss, but also the complex reasons that patients may continue to work.

We also noted inherent socioeconomic disparities in access to employment benefits. Thirteen percent of our study sample reported a complete lack of employment benefits. These respondents were more likely to be older, black, self‐employed, and to report lower annual household income. Our results align with data from the Bureau of Labor Statistics that high‐wage earners and those in professional and managerial occupations are more than twice as likely as low‐wage earners and those with jobs in the service industry to have access to paid sick leave 22. Even among desirable jobs that offer benefits like health insurance and paid sick leave, cancer and its treatment may compound serious illness with personal financial burden 17. For those patients who are at a competitive disadvantage in the marketplace and simultaneously less likely to work for employers who offer benefits, a curable but serious illness such as cancer may be financially devastating. While those patients who are unable to retain their jobs may be eligible to collect unemployment or social security benefits, many are not eligible and those who are receive only a fraction of their previous earnings.

As noted, federal legislation to protect the jobs and health insurance of workers during illness exists as FMLA. Unfortunately, FMLA guarantees only unpaid time off and not all employees are eligible for coverage, even if their employers offer it 8. There have been several attempts to fill the gaps left by FMLA. Statewide paid sick day legislation is in place in four states as well as in the District of Columbia and 18 other municipalities across the United States 23. In September 2015, President Obama issued an Executive Order requiring federal contractors to offer paid sick days to their employees, though this does not extend to Americans working in the private sector 24 and, as an Executive Order, is reversible. Nevertheless, only an estimated one in three workers in the bottom quarter of the pay scale and one in four part‐time workers nationwide have access to paid sick leave 20.

Making policy recommendations regarding employer practices and benefits is difficult. Benefit programs that work well for large employers may be problematic for smaller organizations, especially given the complex state and federal regulations already in place. The costs of workforce turnover or other resources consumed by disruption of organizational stability due to loss of experienced workers are real but complex. In addition, societal costs and benefits associated with such policies have only been preliminarily reported. For example, workers who are able to maintain employment during health shocks rely less on public assistance and contribute more money to their local economies 8, 25.

Our study was subject to several limitations intrinsic to survey research. Analyses were limited by the sample of respondents. We note, however, that the population‐based sampling achieved broad demographic representation and the 68% response rate is higher than any previous published cohort of patients with colorectal cancer 26. The survey relied on respondent report and was thus subject to recall bias, but our reliance on patient reporting permitted individual insights that could not otherwise be obtained. We mitigated recall bias by accepting returned surveys only up until 1 year after diagnosis. Given this relatively short follow‐up period, it is possible that respondents who did not retain their jobs during cancer treatment may have later returned to work. However, previous literature indicates that job loss during cancer treatment, particularly among low‐income patients, predicts long‐term departure from the workforce 27, 28. On the other hand, previous work has found that some cancer patients who temporarily stop working during treatment but then return to work in the first year after diagnosis, as well as some patients who continue to work through treatment and the first year afterward, subsequently quit their jobs for cancer‐related reasons in years 2–5 following diagnosis 29. Therefore, it is also possible that some respondents who retained their jobs may have later departed from the workforce. Because our data were patient‐reported, we did not have access to employer‐level data such as employer size, FMLA availability, or employer‐level benefits. Although we used job retention, defined as working for pay at the time of survey completion, as our primary outcome measure, we acknowledge that there are other measures of employment impacts (decreasing work hours, changing jobs, etc.) among working patients with cancer. We note, however, that job retention reflects the ability to continue to work and earn an income and therefore represents an important element of working patients’ financial well‐being. We did not ask patients who did not retain their jobs whether the work loss was desired or undesired. However, any departure from the labor force reduces a patient's income and may therefore exacerbate the personal financial burden of cancer diagnosis and treatment. There may be unobserved selection bias as we excluded those patients who were not employed at diagnosis from our analyses. Some of these patients could have been previously employed but subsequently chose unemployment based on poor employment benefits or a lack of employment benefits. Finally, although our sample includes patients from rural to urban areas as well as Southern and Midwestern parts of the United States, our data may not be representative of the entire United States.

## Conclusion

Employment benefits, specifically employer‐based health insurance and paid sick leave, were associated with a greater likelihood of job retention during serious illness. Although insurance coverage is on the rise with implementation of the Affordable Care Act, FMLA is more than 20 years old and leaves millions of working Americans potentially unsupported in times of serious illness. Based on our findings, it is plausible that provision of specific employment benefits by employers and government could facilitate job retention and reduce associated financial burden, especially among young working patients.

## Conflicts of Interest

None declared.

## Appendix 1

### Statistical appendix: Propensity score model

**Figure A1.** Distribution of propensity scores by availability of paid sick leave.

Distribution of propensity scores for patients with and without paid sick leave (PSL)

**Table A1:** Propensity quintile bivariates

Covariate	Quintile 1 (%)	Quintile 2 (%)	Quintile 3 (%)	Quintile 4 (%)	Quintile 5 (%)
Age					
<50	11	16	19	27	27
50–64	10	20	24	23	23
65+	65	27	8	0	0
Race					
White	19	21	19	23	19
Black	23	18	22	11	26
Other	31	8	38	23	0
Education					
<High school	56	31	7	7	0
High school grad	28	31	23	7	11
Some college	17	21	22	18	21
College grad	10	10	19	32	28
Gender					
Male	19	25	21	22	14
Female	21	13	19	18	28
Marital					
Not married	27	18	21	13	21
Married	17	21	19	24	20
Income					
<$20K	85	15	0	0	0
$20–$49K	21	33	28	12	6
$50–$89K	8	20	25	21	25
>$90K	7	9	13	35	36
Geographic site					
Detroit	28	25	22	20	5
Georgia	17	18	19	20	26
SES					
Comorbid conditions					
0	15	18	24	32	12
1	18	13	19	16	34
2+	27	29	17	13	14
Job category					
White collar	9	12	16	31	32
Blue collar	35	36	27	3	0
Other	100	0	0	0	0
Employer‐based health insurance					
No	39	26	19	8	8
Yes	13	18	20	25	25
Paid sick leave					
No	5	18	19	28	30
Yes	37	25	21	9	8
Extended sick leave					
No	7	15	21	28	28
Yes	31	25	19	11	13
Unpaid time off					
No	12	19	21	24	24
Yes	25	23	20	15	17
Disability benefits					
No	5	19	21	30	24
Yes	30	22	20	11	17

**Table A2:** Sociodemographic variables and paid sick leave in the propensity‐adjusted model

Covariate	Before adjustment	After adjustment
Age	<.0001	0.7730
Race	0.8527	0.8209
Education	0.0002	0.9790
Gender	0.3302	0.7861
Marital status	0.4004	0.6806
Income	<.0001	0.9124
Geographic site	0.0319	0.9971
SES	0.0145	0.9010
Comorbid conditions	0.1300	0.8609
Job category	<.0001	0.8929
